# Employing AI-driven predictive analytics to enhance patient outcomes and streamline healthcare processes

**DOI:** 10.3389/frai.2026.1947926

**Published:** 2026-09-28

**Authors:** Justin Pathrose James

**Affiliations:** LA Care Health Plan, Los Angeles, CA, United States

**Keywords:** analytics, artificial intelligence, data analytics, forecast accuracy, healthcare, predictive modeling

## Abstract

Artificial intelligence (AI) can advance healthcare analytics by generating predictive insights that extend beyond traditional retrospective reporting. This study examines how machine learning models applied to clinical, operational, and contextual data may identify patient risk factors, forecast disease progression, and support timely interventions. Rather than claiming definitive clinical impact, the manuscript presents an implementation-oriented framework showing how predictive models can inform readmission risk stratification, resource planning, and care quality improvement when supported by rigorous validation, explainability, ethical governance, and transparent data practices. Case-based discussion and a reproducible analytic workflow illustrate how AI-driven predictive analytics may be translated into decision support across healthcare settings.

## Introduction

Healthcare executives face persistent pressure to reduce patient dissatisfaction, prevent avoidable readmissions, and improve outcomes despite limited financial resources. AI-driven predictive analytics offers a practical response by identifying risks earlier, streamlining care processes, and revealing opportunities for organizational growth. This opening context frames the manuscript’s central question: how can predictive models be structured so that statistical outputs become clinically meaningful and operationally useful? The following sections move from this rationale to the analytic workflow, implementation considerations, limitations, and future research needs.

Machine learning has emerged as a foundational technology in modern predictive analytics, enabling healthcare organizations to shift from retrospective reporting to proactive, data-driven decision-making. Advances in deep learning allow models to extract clinically meaningful patterns from complex, high-dimensional data, including imaging, free-text notes, and longitudinal electronic health record data. Building on this foundation, subsequent studies have demonstrated the value of predictive models in forecasting adverse events, such as sepsis, readmissions, and disease progression, often outperforming traditional statistical approaches. Research in operational settings further indicates that AI-based forecasting can improve staffing, bed utilization, and resource allocation, underscoring the technology’s relevance beyond direct clinical care. Collectively, these studies position AI-driven predictive analytics as a transformative but still maturing capability that requires rigorous governance and real-world validation to achieve sustainable impact (Rajkomar et al., 2019).

## AI-driven predictive analytics in healthcare

Figure 1 summarizes the logic underlying the manuscript: heterogeneous healthcare data are processed through AI models to generate insights that can inform patient outcomes and operational decisions.

**Figure 1:**
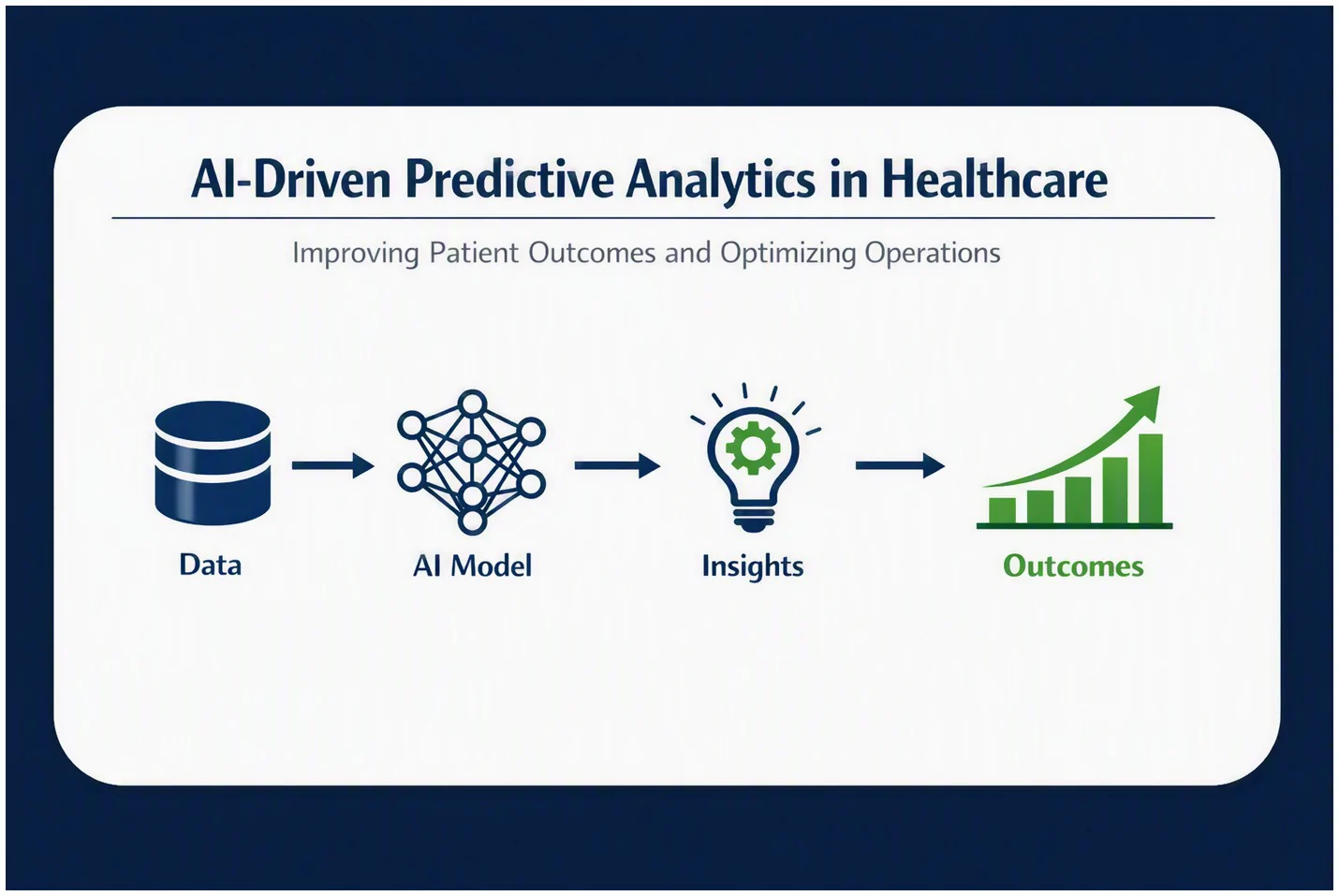
AI-driven predictive analytics workflow in healthcare, illustrating the progression from data acquisition to AI modeling, insight generation, and improved operational and patient outcomes.

### Problem context

Building on this workflow, the problem context begins with the operational pressures that make predictive analytics relevant. Healthcare organizations operate under intersecting constraints, including rising costs, limited staffing and financial resources, growing demand for personalized care, and the need to make decisions from increasingly complex clinical and operational datasets.

The increasing digitization of healthcare has created substantial opportunities to forecast patient outcomes with greater accuracy and scale. Limitations in traditional clinical heuristics and conventional risk scores create space for machine learning models to detect nonlinear patterns and subtle interactions that may be difficult to identify in time-constrained or complex clinical contexts. Predictive analytics therefore represents not merely a technical enhancement but a structural shift in how healthcare systems identify risk, allocate resources, and support earlier interventions across the disease trajectory. Subsequent empirical work has supported this view, demonstrating stronger performance from machine learning approaches in predicting outcomes such as heart failure exacerbations, sepsis onset, and hospital readmissions. However, the literature also emphasizes persistent challenges related to data bias, transparency, and the potential for automated decision systems to reinforce existing inequities (Obermeyer and Emanuel, 2016).

### The AI advantage

In response to these pressures, AI-driven predictive analytics may add value by identifying risk patterns before clinical deterioration, supporting more targeted resource allocation, and providing timely decision support for assessment, intervention, and individualized care planning.

### Key applications

These advantages translate into several practical applications. Key uses include population health management, readmission-risk prediction, operational forecasting, real-time monitoring through connected devices, and explainable decision support. Across these applications, the purpose of AI is to convert large volumes of heterogeneous data into interpretable signals that can guide prevention, scheduling, follow-up, and care coordination.

The literature indicates that predictive analytics is most useful when large, diverse, and high-quality datasets are paired with rigorous methodological standards. Although larger datasets can improve model development, they also introduce risks related to data heterogeneity, missingness, and spurious associations if models are not carefully validated. Transparent evaluation frameworks are therefore essential because predictive models must demonstrate not only statistical performance, but also clinical usefulness and reproducibility across settings (Beam and Kohane, 2018).

### Ethical and governance considerations

Because predictive analytics can influence clinical decisions, resource allocation, and patient access to services, ethical governance must be treated as a core implementation requirement rather than a secondary consideration. Responsible deployment requires systematic attention to fairness, bias mitigation, patient privacy, regulatory compliance, transparency, and accountability across the AI lifecycle.

Governance processes should begin with the data used to develop and validate predictive models. Clinical and administrative datasets may contain historical inequities, inconsistent documentation practices, and uneven representation across patient groups. Without appropriate safeguards, AI systems may reproduce these patterns in ways that disadvantage already underserved populations. Model development should therefore include data-quality assessment, subgroup performance evaluation, fairness auditing, and procedures for identifying whether predictions differ systematically across clinically relevant populations.

Transparency and accountability are equally important. Clinicians and administrators need sufficient explanation to understand why a model assigns risk, which variables influence predictions, and how uncertainty should be interpreted. Explainability tools can support this process, but they do not eliminate the need for human oversight, clear escalation procedures, and documented responsibility for AI-informed decisions. Governance should also include ongoing monitoring for model drift, periodic recalibration, privacy protections, secure data access controls, and review mechanisms that ensure predictive analytics remains aligned with clinical judgment, institutional policy, and patient-centered care (Goldstein et al., 2017).

### Expected impact

Taken together, the conceptual framework, empirical literature, and governance considerations suggest that the impact of AI-driven predictive analytics should be understood as conditional rather than automatic. Its primary value lies in strengthening risk stratification, supporting earlier care management interventions, improving resource planning, and enabling organizational learning from complex healthcare data. However, these benefits depend on the quality and representativeness of the data, the calibration and external validation of predictive models, the interpretability of model outputs, and the extent to which clinicians can act on timely and trustworthy information. Accordingly, AI-driven predictive analytics is most likely to improve healthcare processes when it is embedded within accountable clinical workflows and paired with interventions capable of changing patient outcomes.

## Methods

### Study design

To examine how such conditional benefits might be pursued, this study was conceptualized as an applied, exploratory analysis of AI-driven predictive analytics in healthcare. The research was structured to examine how machine learning techniques can be used to transform clinical, operational, and patient-generated data into actionable predictions that support improved patient outcomes and more efficient healthcare processes. The design integrated a literature-informed conceptual framework with a practical implementation model focused on 30-day hospital readmission risk, allowing the study to connect theoretical discussion with an applied predictive analytics workflow.

The methodological structure emphasized three related dimensions: data acquisition, model development, and outcome translation. Data acquisition focused on the use of structured and unstructured information from electronic health records, wearable devices, Internet of Things systems, and administrative sources. Model development centered on the application of machine learning methods to identify patient risk patterns and forecast clinically relevant outcomes. Outcome translation addressed how predictive outputs could be incorporated into dashboards, care management workflows, and administrative decision-making processes.

### Data analysis

Within this design, data analysis was conducted using a supervised machine learning framework designed to predict 30-day hospital readmission risk. The analytical process began with data preprocessing to improve consistency and model readiness. Numeric variables were imputed using median values and standardized to support stable model estimation, while categorical variables were imputed using the most frequent category and transformed through one-hot encoding. The dataset was then partitioned into training and testing subsets to support out-of-sample evaluation and reduce the likelihood of overfitting.

Predictive modeling was performed using a gradient boosting classifier, selected because ensemble-based methods can capture nonlinear relationships and interaction effects commonly present in clinical and operational datasets. Probability calibration was applied to improve the interpretability and practical usefulness of predicted risk scores. Model performance was assessed using discrimination and classification metrics, including ROC-AUC, precision-recall AUC, confusion matrix outputs, and classification reporting. These measures provided complementary evidence regarding the model’s ability to distinguish high-risk patients, manage class imbalance, and support threshold-based clinical decision-making.

Additional analytical procedures focused on interpretability and operational translation. Feature importance and SHAP-based explanations were used to identify variables contributing most strongly to predicted readmission risk at both global and patient-specific levels. Predicted probabilities were converted into risk tiers and care management flags to demonstrate how analytical outputs could be integrated into dashboards and clinical workflows. Together, these approaches linked statistical model performance with practical decision support for population health management, resource allocation, and targeted intervention planning.

### Study execution

After defining the analytical approach, the study execution focused on translating the conceptual framework into an implementation-oriented workflow. The research was conducted by first identifying key healthcare challenges that predictive analytics is positioned to address, including avoidable readmissions, resource constraints, care coordination gaps, and the need for timely risk detection. Relevant literature was reviewed to establish the theoretical foundation for AI-based forecasting in healthcare and to identify recurring concerns related to model accuracy, transparency, bias, governance, and clinical integration. These findings informed the development of a practical analytics workflow that reflects common stages in predictive modeling.

The implementation component was organized around a supervised machine learning approach for predicting 30-day hospital readmission. The workflow included data loading, preprocessing, train-test partitioning, model training, probability calibration, performance evaluation, interpretability analysis, and operational output generation. Preprocessing procedures accounted for missing values, categorical encoding, and feature scaling. Model performance was evaluated using standard classification metrics, including ROC-AUC, precision-recall AUC, confusion matrix outputs, and classification reporting. Explainability methods, such as feature importance and SHAP-based interpretation, were incorporated to support transparency and clinician trust.

Finally, the execution framework translated model outputs into practical healthcare actions by generating risk scores, assigning risk tiers, and producing care management flags for dashboard or workflow integration. This approach illustrates how predictive analytics can move beyond statistical prediction toward operational decision support. The study therefore combines conceptual analysis with implementation-oriented modeling to demonstrate how AI can be responsibly applied to healthcare growth, patient risk management, and process improvement.

## Results and experimental analysis

Following the methods, the results section explains the purpose and contribution of each stage in the experimental workflow. The workflow was designed to demonstrate how predictive analytics can move from raw healthcare data to actionable clinical and operational outputs. The data preparation experiment established whether heterogeneous clinical and administrative variables could be standardized into an analyzable format. This step was necessary because predictive accuracy and clinical reliability depend on consistent handling of missing values, categorical variables, and feature scaling.

The model development experiment then evaluated whether a supervised machine learning approach could distinguish patients at higher risk of 30-day readmission from those at lower risk. The use of gradient boosting was intended to capture nonlinear relationships among demographic, utilization, clinical, and social variables. Its purpose was not only to generate a prediction, but also to show how multivariable patterns can support earlier identification of patients who may benefit from targeted follow-up.

The evaluation experiment assessed the practical usefulness of the model through complementary performance measures. ROC-AUC examined the model’s overall ability to discriminate between readmitted and non-readmitted patients, while precision-recall AUC was especially relevant for evaluating performance when positive cases may be less frequent. The confusion matrix and classification report provided threshold-specific evidence about false positives, false negatives, precision, recall, and the trade-offs that healthcare organizations must consider when using predictive scores to guide intervention decisions.

The interpretability experiment contributed an additional layer of analysis by identifying which features had the greatest influence on model predictions. Global feature-importance and SHAP-based explanations helped connect model outputs to clinically meaningful variables, while patient-level explanations illustrated how individual risk scores could be interpreted in practice. This component was essential because predictive performance alone is insufficient for healthcare deployment; clinicians must be able to understand why a model assigns risk and whether that reasoning is clinically plausible.

Finally, the operationalization experiment demonstrated how predicted probabilities could be converted into risk tiers and care management flags. This stage clarified the practical contribution of the analytics pipeline by translating statistical outputs into workflow-ready information for dashboards, population health teams, and discharge planning processes. Together, the experiments show that AI-driven predictive analytics contributes most effectively when model development, interpretability, and implementation planning are treated as integrated components rather than isolated technical tasks.

### Implementation example

To make the preceding workflow reproducible, the following code example operationalizes the methods and analysis through an end-to-end readmission-risk prediction pipeline. It is included as an illustrative implementation rather than as evidence from a completed clinical trial.


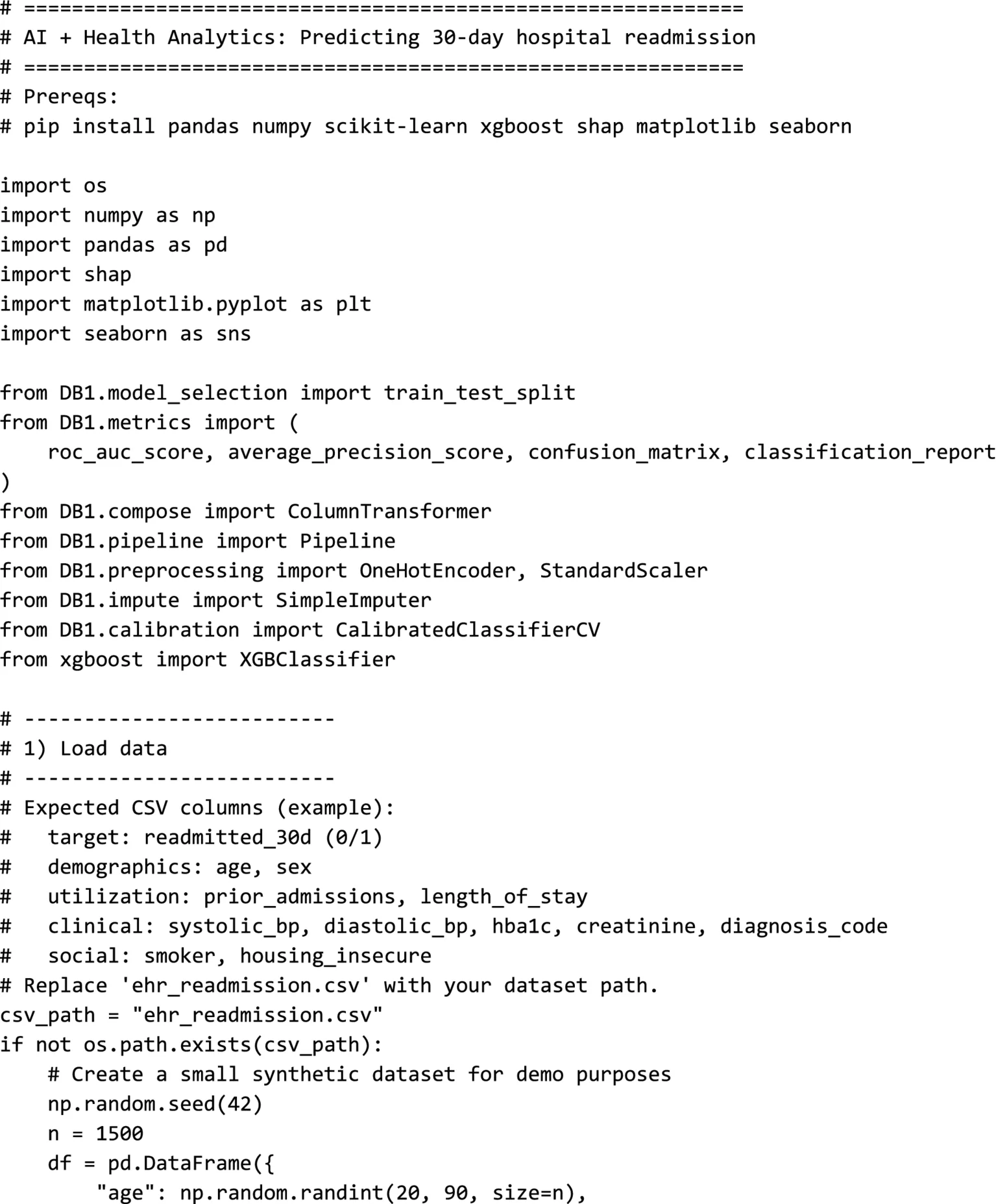



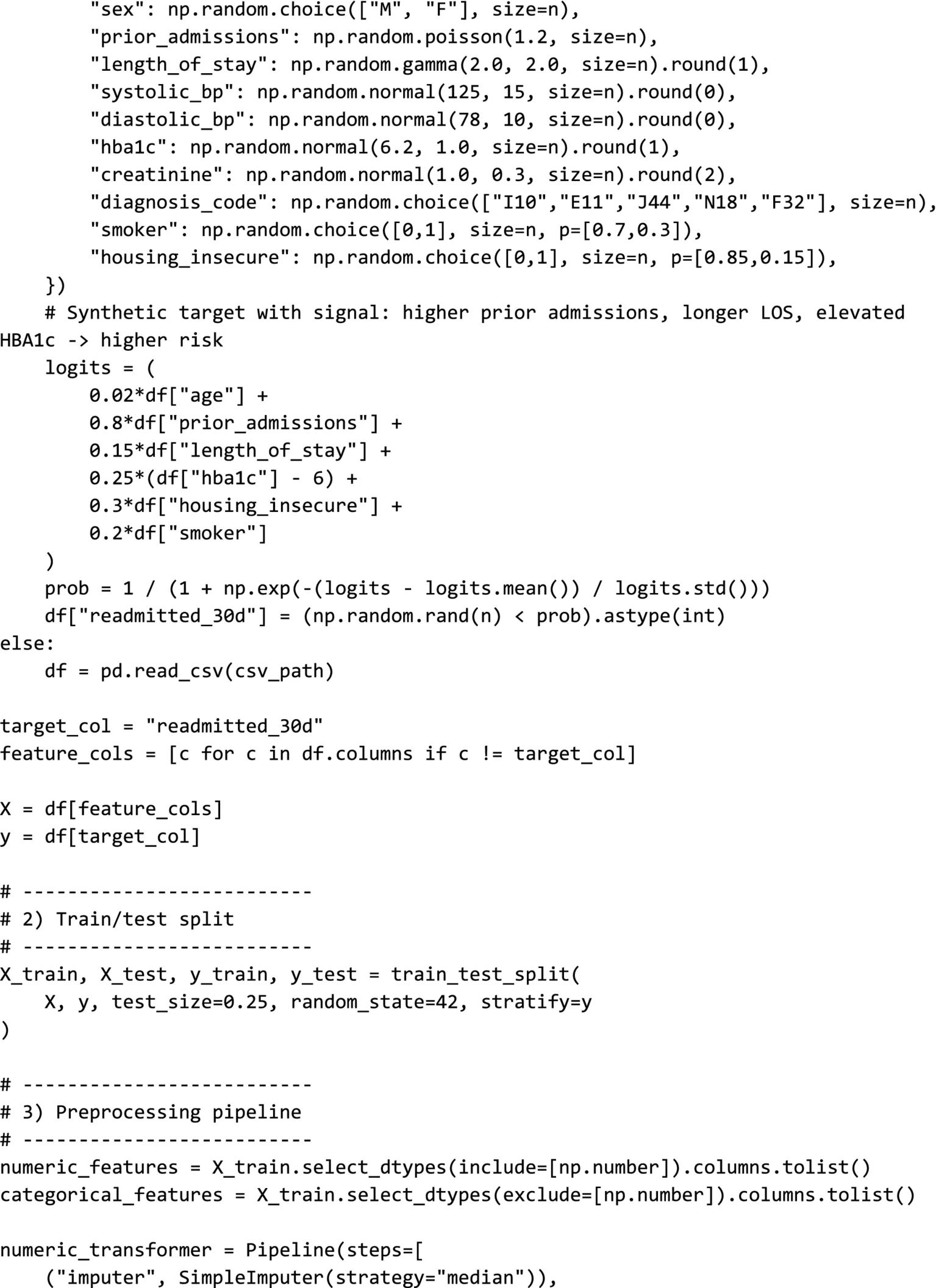



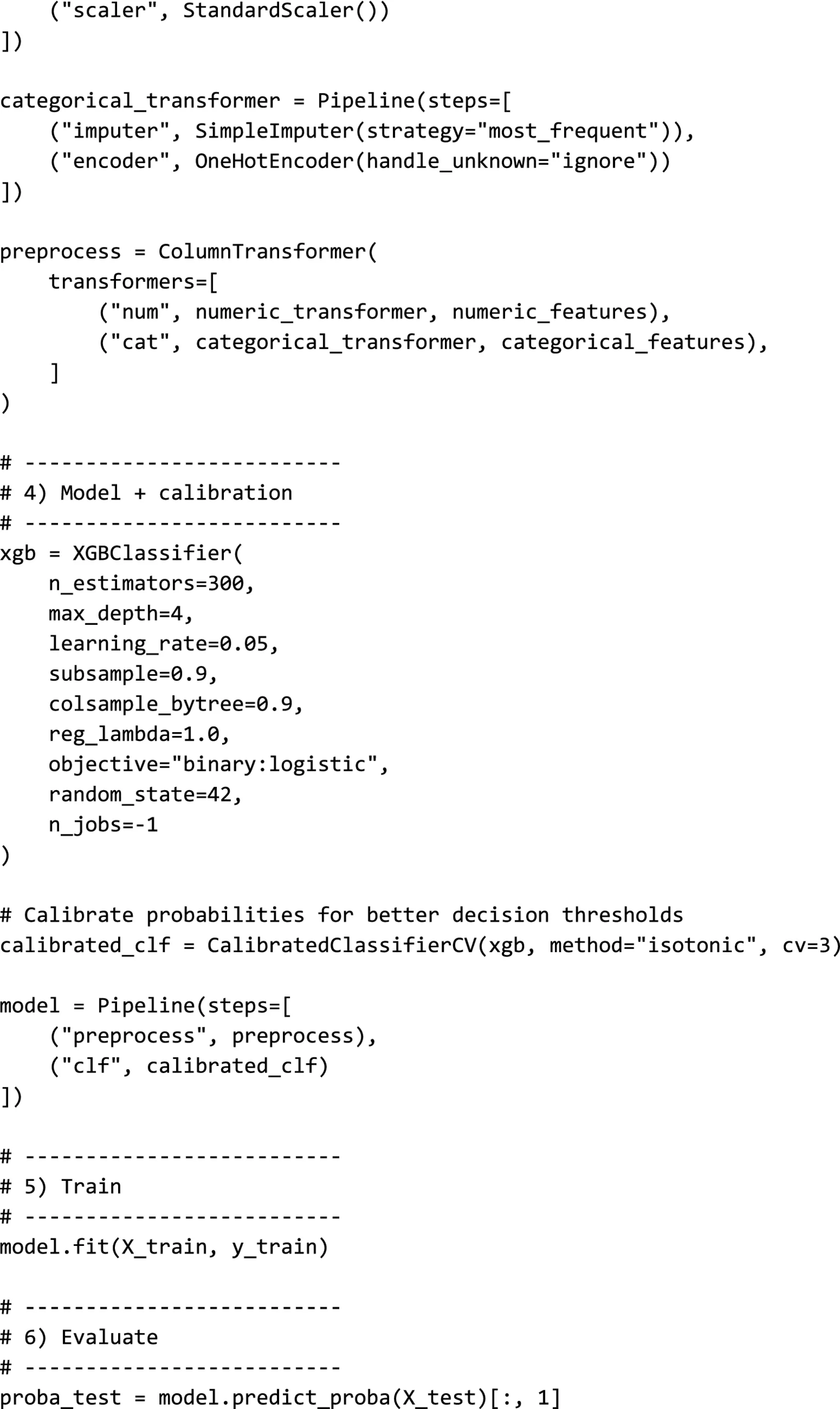



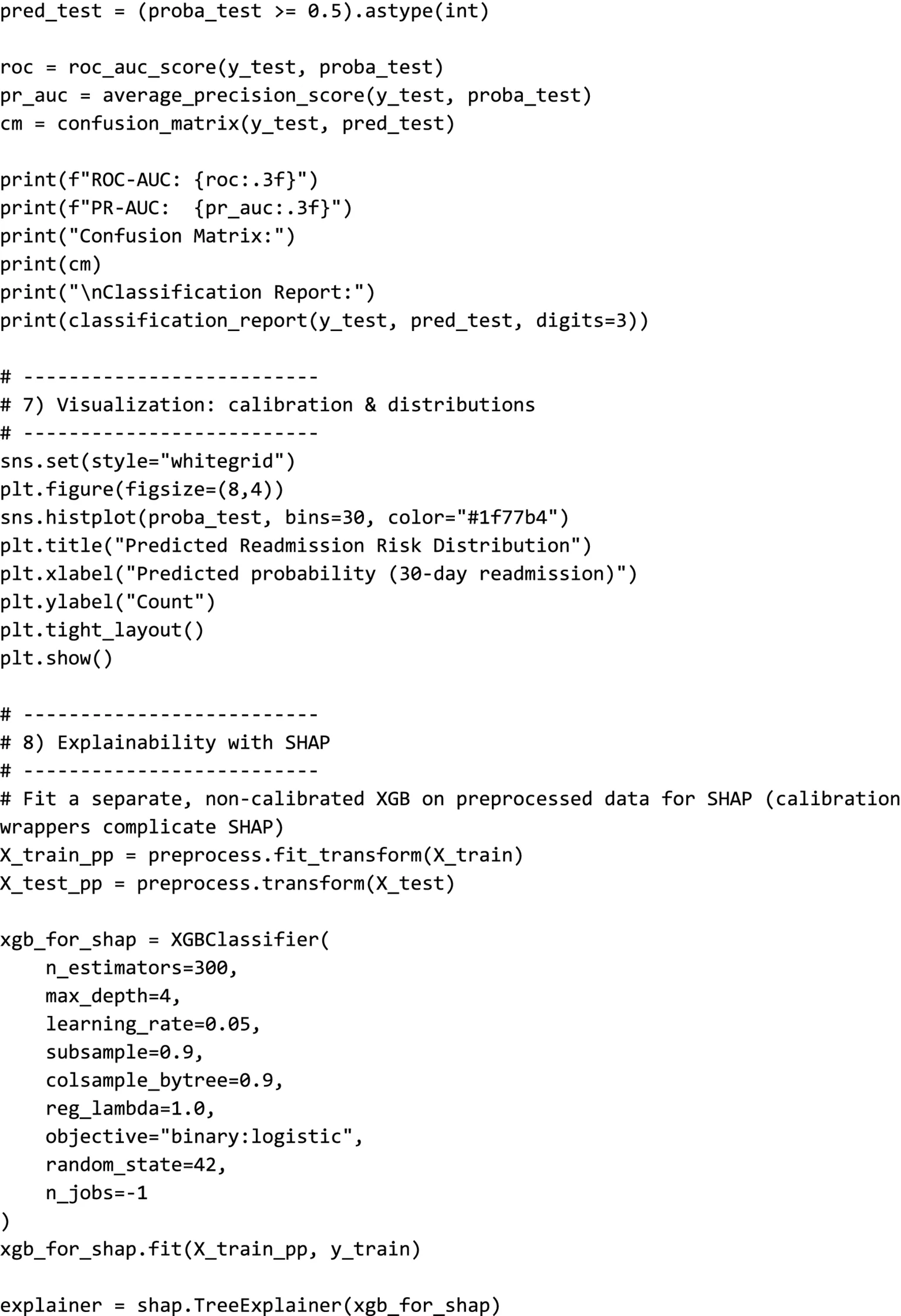



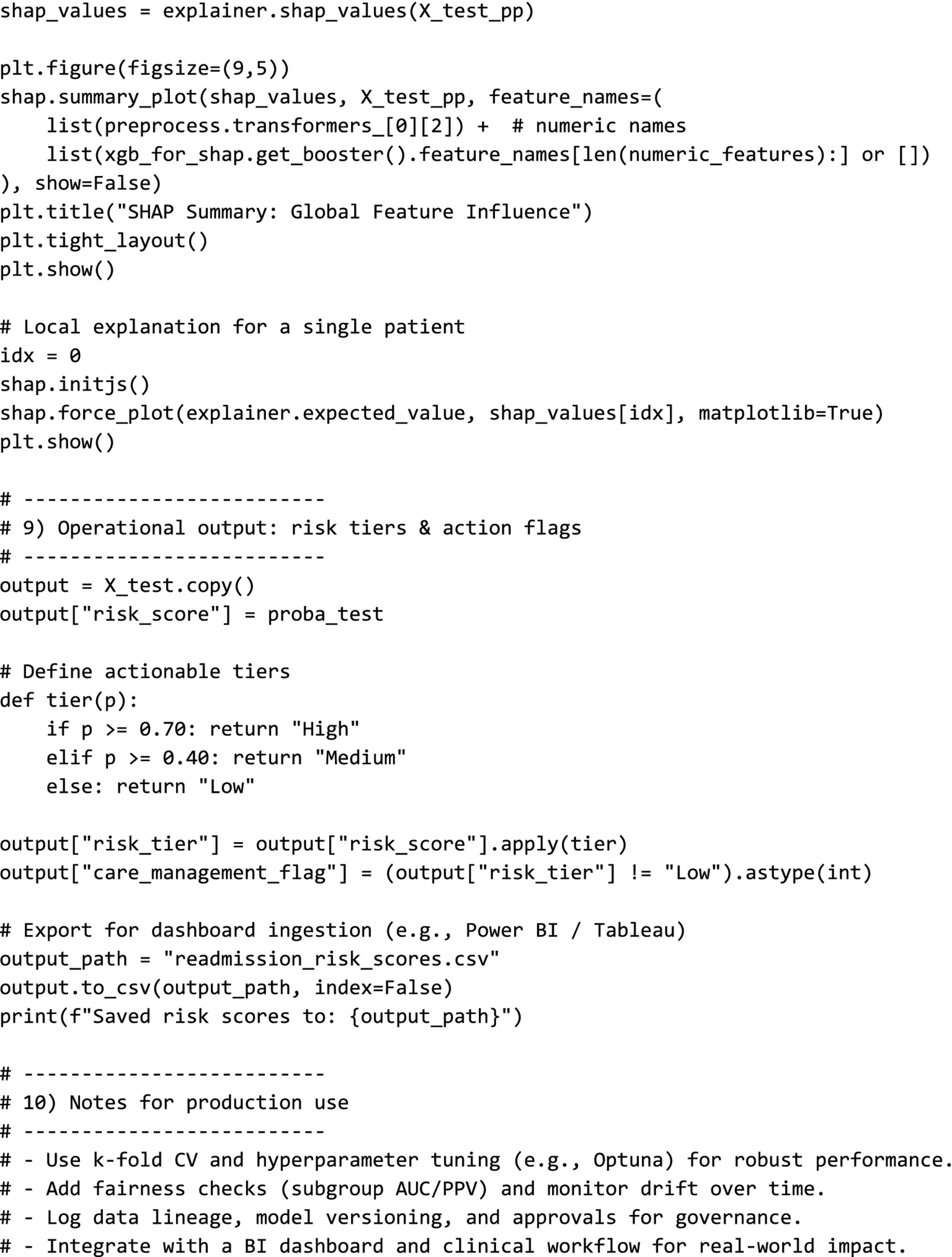


## Limitations of AI in predictive analytics

The implementation example also underscores why limitations must be considered before clinical deployment. Despite its potential to improve healthcare decision-making, AI-driven predictive analytics depend heavily on the quality, completeness, and representativeness of the underlying data. Electronic health records, wearable devices, and administrative datasets often contain missing values, inconsistent documentation practices, coding errors, and uneven representation across patient populations. As a result, predictive models may produce unreliable or inequitable outputs when trained on incomplete or biased datasets.

Second, AI systems may reinforce or amplify existing disparities in healthcare delivery. If historical data reflect unequal access to care, differences in documentation, or biased treatment patterns, the algorithm may learn these patterns and reproduce them in future predictions. This limitation is particularly concerning in clinical contexts where risk scores influence resource allocation, follow-up intensity, or eligibility for preventive interventions.

Third, many advanced AI models remain difficult to interpret. Although complex machine learning methods may achieve strong predictive accuracy, their internal logic can be opaque to clinicians, administrators, and patients. Limited interpretability can reduce trust, complicate clinical accountability, and make it difficult to determine whether a prediction is based on clinically meaningful variables or on misleading correlations.

Fourth, predictive performance may decline when models are applied outside the settings in which they were developed. Differences in patient demographics, clinical workflows, technology infrastructure, documentation norms, and treatment protocols can reduce model generalizability. In addition, model drift may occur over time as disease patterns, care practices, or population characteristics change, requiring continuous monitoring, recalibration, and validation.

Finally, AI implementation raises privacy, security, workflow, and governance challenges. Predictive analytics relies on sensitive patient information, requiring strong safeguards for data access, consent, storage, and regulatory compliance. Even technically accurate models may fail to improve outcomes if they are not integrated effectively into clinical workflows or if clinicians experience alert fatigue, automation bias, or uncertainty about responsibility for AI-informed decisions. Therefore, AI should be understood as a decision-support tool rather than a substitute for professional judgment, ethical oversight, and patient-centered care.

## Conclusion and future research

In light of these limitations, this manuscript supports the conclusion that AI-driven predictive analytics can strengthen healthcare risk stratification when implemented with appropriate methodological safeguards. The review and implementation framework indicate that machine learning approaches are well suited to identifying multivariable risk patterns in readmission prediction, particularly when clinical, operational, and contextual data are integrated. However, the contribution of this study is best understood as conceptual and implementation-oriented: it demonstrates how predictive analytics can be structured, evaluated, interpreted, and translated into workflow-ready outputs, rather than proving direct reductions in readmissions or costs.

The conclusions should therefore be interpreted cautiously. Improved prediction does not automatically produce improved outcomes unless models are externally validated, calibrated for the intended population, embedded into clinical workflows, and paired with effective interventions. Future research should use larger, multi-institutional datasets, compare model families under consistent validation protocols, and evaluate whether AI-supported decisions measurably improve readmission rates, care coordination, patient experience, and cost efficiency in real-world healthcare environments.

Future research should also strengthen the empirical basis for implementation by conducting longitudinal evaluations, external validation studies, and prospective impact assessments. Longitudinal studies are needed to determine whether performance declines because of model drift, changes in patient populations, or shifts in clinical practice. External validation should examine whether models trained in one health system remain accurate when applied to different institutions, demographic groups, and documentation environments. Prospective studies should evaluate not only statistical metrics, such as ROC-AUC, precision-recall AUC, calibration, sensitivity, and specificity, but also implementation outcomes, including clinician adoption, alert burden, equity across subgroups, patient satisfaction, readmission rates, and cost-effectiveness. These forms of evidence would provide a stronger foundation for determining when AI-driven predictive analytics produces measurable improvements in patient care rather than simply generating technically accurate predictions.
